# IJCARS: BVM 2021 special issue

**DOI:** 10.1007/s11548-021-02534-7

**Published:** 2021-11-18

**Authors:** Andreas Maier, Thomas M. Deserno, Heinz Handels, Klaus Maier-Hein, Christoph Palm, Thomas Tolxdorff

**Affiliations:** 1 Pattern Recognition Lab, SAOT Graduate School in Advanced Optical Technologies, Friedrich-Alexander-University Erlangen-Nürnberg, Martensstr. 3, 91058 Erlangen, Germany; 2 Peter L. Reichertz Institute for Medical Informatics, Technical University of Braunschweig, Muehlenpfordtstr. 23, 38106 Brunswick, Germany; 3 Sektion Naturwissenschaften, Institut für Medizinische Informatik, Universität zu Lübeck, Ratzeburger Allee 160, 23538 Lübeck, Germany; 4 Division of Medical Image Computing (E230), Deutsches Krebsforschungszentrum, Im Neuenheimer Feld 223, 69120 Heidelberg, Germany; 5 Medical Image Computing (Re-MIC), Faculty of Computer Science and Mathematics, Regensburg University of Applied Sciences, Galgenbergstr. 32, 93053 Regensburg, Germany; 6 Institut für Medizinische Informatik, Biometrie und Epidemiologie, Charité - Universitätsmedizin Berlin, Campus Benjamin Franklin, Hindenburgdamm 30, 12200 Berlin, Germany

The German workshop on medical image computing (BVM) has been held in different locations in Germany for more than 20 years. In terms of content, BVM focused on the computer-aided analysis of medical image data with a wide range of applications, e.g. in the area of imaging, diagnostics, operation planning, computer-aided intervention and visualization.

During this time, there have been remarkable methodological developments and upheavals, on which the BVM community has worked intensively. The area of machine learning should be emphasized, which has led to significant improvements, especially for tasks of classification and segmentation, but increasingly also in image formation and registration. As a result, work in connection with deep learning now dominates the BVM. These developments have also contributed to the establishment of medical image processing at the interface between computer science and medicine as one of the key technologies for the digitization of the health system.

In addition to the presentation of current research results, a central aspect of the BVM is primarily the promotion of young scientists from the diverse BVM community, covering not only Germany but also Austria, Switzerland, The Netherland and other European neighbors. The conference serves primarily doctoral students and postdocs, but also students with excellent bachelor and master theses as a platform to present their work, to enter into professional discourse with the community, and to establish networks with specialist colleagues. Despite the many conferences and congresses that are also relevant for medical image processing, the BVM has therefore lost none of its importance and attractiveness and has retained its permanent place in the annual conference rhythm.

Building on this foundation, there are some innovations and changes this year. The BVM 2021 was organized for the first time at the Ostbayerische Technische Hochschule Regensburg (OTH Regensburg, a technical university of applied sciences). After Aachen, Berlin, Erlangen, Freiburg, Hamburg, Heidelberg, Leipzig, Lübeck, and Munich, Regensburg is not just a new venue. OTH Regensburg is the first representative of the universities of applied sciences (HAW) to organize the conference, which differs to universities, university hospitals, or research centers like Fraunhofer or Helmholtz. This also considers the further development of the research landscape in Germany, where HAWs increasingly contribute to applied research in addition to their focus on teaching. This development is also reflected in the contributions submitted to the BVM in recent years.

At BVM 2021, which was held in a virtual format for the first time due to the Corona pandemic, an attractive and high-quality program was offered. Fortunately, the number of submissions increased significantly. Out of 97 submissions, 26 presentations, 51 posters and 5 software demonstrations were accepted via an anonymized reviewing process with three reviews each. The three best works have been awarded BVM prizes, selected by a separate committee.

Based on these high-quality submissions, we are able to present another special issue in the International Journal of Computer Assisted Radiology and Surgery (IJCARS). Out of the 97 submissions, the ones with the highest scores have been invited to submit an extended version of their paper to be presented in IJCARS. As a result, we are now able to present this special issue with seven excellent articles. Many submissions focus on machine learning in a medical context.

Denck et al. present a method to use generative adversarial networks to virtually map magnetic resonance images into different contrasts by specifying different repetition and echo times with respect to the acquisition pulse sequence. The results indicate that the virtually mapped images are close to images that were acquired on a real scanner.

Bigalke et al. present a method to estimate the weight of a patient lying under a blanket in the operation room. It is based on a 3D U-Net which is used to virtually remove the cover. Next, another deep network is used to predict the weight of the patient. The results show that the weight estimation only differs by approximately 5 kg from the true patient weight.

Image reconstruction using deep networks has been criticized for being unreliable in some situations. Therefore, Oala et al. propose a novel approach to detect failures thereof using interval neural network-based uncertainty estimation. As the experiments indicate, their approach is superior to several state-of-the-art uncertainty estimation methods such as Monte Carlo Dropout.

Reconstruction artifacts also play an important role in the paper by Felsner et al. In particular, sensitivity maps are important to correct inhomogeneous phase distributions. In the article, an iterative, a known operator, and a pure deep learning method are compared in detail and advantages and disadvantages of each method are discussed.

Detection of sutures in endoscopy can support real-time tracking and analysis of the images. Therefore, Sharan et al. present a deep learning-based approach using heatmap regression to detect the suture points. In their results, they can demonstrate that their deep learning approach is superior to other baseline approaches.

Dünnwald et al. present a fully automatic method to locate and segment the locus coeruleus in patients with Parkinson’s disease. In their work, they successfully apply a U-Net-based approach to tackle the problem. On average, the algorithm is only about 2 mm off in the localization task. The segmentation is also robust with a Dice similarity of 71%.

Intracranial arteriovenous malformations (AVMs) show complex anatomy. This in particular poses a challenge for treatment via embolization. Therefore, Sprengel et al. propose a method to virtually predict the outcome of the treatment which will allow physicians to explore different treatment options. In their results, they demonstrate that such a feature is perceived as highly beneficial by experienced radiologists.

As guest editors of this IJCARS-BVM special issue, we are very proud to be able to present so many high-quality papers and congratulate the authors to their success. Also, the BVM awards for the best contributions could be awarded again this year at the virtual workshop. This is a clear indication of the strong research environment in the BVM community and we hope that we will also see more exciting research here in the years to come!

